# Platelet Inflammatory Response to Stress

**DOI:** 10.3389/fimmu.2019.01478

**Published:** 2019-06-28

**Authors:** Fabrice Cognasse, Sandrine Laradi, Philippe Berthelot, Thomas Bourlet, Hubert Marotte, Patrick Mismetti, Olivier Garraud, Hind Hamzeh-Cognasse

**Affiliations:** ^1^Etablissement Français du Sang Auvergne-Rhône-Alpes, Saint-Étienne, France; ^2^GIMAP-EA3064, Université de Lyon, Saint-Étienne, France; ^3^Laboratoire des Agents Infectieux et d'Hygiène, CHU de Saint-Etienne, Saint-Étienne, France; ^4^SAINBIOSE, INSERM U1059, University of Lyon, Saint-Étienne, France; ^5^Department of Rheumatology, University Hospital of Saint-Etienne, Saint-Étienne, France; ^6^Vascular and Therapeutic Medicine Department, Saint-Etienne University Hospital Center, Saint-Étienne, France; ^7^Institut National de Transfusion Sanguine, Paris, France

**Keywords:** platelets, innate immunity, transfusion, cytokine/chemokine, inflammation

## Abstract

Blood platelets play a central hemostatic role, (i) as they repair vascular epithelial damage, and (ii) they play immune defense roles, as they have the capacity to produce and secrete various cytokines, chemokines, and related products. Platelets sense and respond to local dangers (infectious or not). Platelets, therefore, mediate inflammation, express and use receptors to bind infectious pathogen moieties and endogenous ligands, among other components. Platelets contribute to effective pathogen clearance. Damage-associated molecular patterns (DAMPs) are danger signals released during inflammatory stress, such as burns, trauma and infection. Each pathogen is recognized by its specific molecular signature or pathogen-associated molecular pattern (PAMP). Recent data demonstrate that platelets have the capacity to sense external danger signals (DAMPs or PAMPs) differentially through a distinct type of pathogen recognition receptor (such as Toll-like receptors). Platelets regulate the innate immune response to pathogens and/or endogenous molecules, presenting several types of “danger” signals using a complete signalosome. Platelets, therefore, use complex tools to mediate a wide range of functions from danger sensing to tissue repair. Moreover, we noted that the secretory capacity of stored platelets over time and the development of stress lesions by platelets upon collection, processing, and storage are considered stress signals. The key message of this review is the “inflammatory response to stress” function of platelets in an infectious or non-infectious context.

## Introduction

Several reviews have recently been published discussing the role of the interaction between platelets and both vascular endothelial cells and leukocytes during hemostasis and the initiation of the vascular repair process (1–3). This review focuses on the interactions between platelets and their environment beyond hemostasis, particularly in inflammation. It has been suggested that platelets detect and respond to local dangers such as infectious pathogens accidentally introduced into the bloodstream at the site of wounds. To achieve both goals (hemostasis /vascular repair and danger sensing), platelets use both membrane-bound and secreted products that interact with other cell types, including leukocytes.

## Platelets as Key Players in Inflammation

Although platelets are regarded primarily as cells associated with hemostasis, it has now become clear that platelets play a wide variety of roles. Endothelial wall alteration or disruption exposes the sub-endothelial matrix rich in prohemostatic proteins. The engagement of platelet surface receptors with these matrix proteins leads to (i) platelet adhesion at the sites of lesions and (ii) the initiation of a complex intracellular signaling process (4). This process results in the formation and release of transcellular mediators, the exocytosis of adhesive and inflammatory proteins and the expression of both additional adhesive receptors and a procoagulant surface (5). Developing platelets retain cytoplasmic granules from their precursors during megacaryocyte differentiation and platelet production, the contents of which are secreted during platelet activation. Exocytosis of the platelet granule contents requires granule membranes to be fused with plasma membranes or open canalicular system (OCS) membranes. The OCS provides a transportation pathway for the release of platelet granule contents (6, 7). In general, these contents fall into three types. First, dense (δ) granules, which are rich in ADP, ATP, calcium, and serotonin, play an important role during hemostasis. Second are alpha (α) granules, which contain a variety of proteins, including adhesive proteins such as thrombospondin, von Willebrand factor, and fibronectin; growth factors such as insulin-like growth factor (IGF), transforming growth factor beta (TGF-β), and platelet-derived growth factor (PDGF); platelet factor 4 (PF4); and a variety of pro-inflammatory/modulatory chemokines and cytokines (1). In addition, α-granule exocytosis results in the expression of P-selectin (CD62P) on the external surface of platelets (5). Interestingly, there is now sound evidence to support the fact that α-granules are heterogeneous, in terms of both their contents and their exocytotic regulation (8–10). Third are platelet-enclosed lysosomes, which secrete hydrolases after activation (11). More recent reports have described a possible new type of granule termed a T-granule (11, 12). Resting platelets express basal surface TLR9 levels that increase significantly after thrombin activation, suggesting that although the majority of TLR9 is expressed intracellularly, some is relocalized to the plasma membrane upon agonist exposure (11–13).

The influence of the cytoskeleton on granule secretion has been a matter of discussion, with studies suggesting that reorganization of microtubules does not affect granule secretion (14, 15). Other studies suggest that the cytoskeleton does not facilitate the secretion of granules and that F-actin disassembly may be required for normal secretion of granules (16). In addition, the function of α-granules is dependent on inflammation, atherosclerosis, angiogenesis, wound healing, antimicrobial host defense, and platelet function in malignant hematological disorders. However, there is little knowledge about the cellular processes that help platelets release α-granule contents. Kamykowski et al. attributed the segregation of α-granule contents previously observed by investigators to the compartmentalization of cargo within single α-granules (17), while other reports showed that angiogenic factors are localized to different α-granules and released by different agonists upon stimulation (18, 19). Lastly, van Nispen tot Pannerden et al. showed that high spatial protein gradients exist within platelet α-granules and propose that tubular α-granules have different secretory capacities than conventional spherical granules and that the spatial segregation of cargo within tubular subtypes may result in differential release of their contents (20). A greater understanding of the dynamics of the fusion pore may illuminate the ways in which platelets drive the release of granule content with disparate platelet functions.

It has been known for more than three decades that platelets release arachidonic acid from membrane phospholipids (21). Arachidonic acid is converted into thromboxane A2 (TxA2), which has both prothrombotic and vasoconstrictive properties. This pathway serves as the target for aspirin, the primary antithrombotic currently in use. As outlined above, if an injured vessel is exposed to subendothelial structures (e.g., collagen), circulating platelets respond quickly, convert to an activated state, adhere and start to form the characteristic hemostatic clot (3). Among other processes, repair of the damaged vessel involves activated endothelial cells. Several reports describe the involvement of various cell adhesion molecules (e.g., P-Selectin, GPIb, GPVI, GPIaβ1, GPIIbβ3, CD40L, TNSF14; JAM-A, PSGL-1, P-Selectin, αvβ3, ICAM-1, CD40, TNSF14R, and JAM-A) acting at the interface between platelets and endothelial cells (19, 22, 23). P-selectin is a well-characterized endothelial and platelet adhesion receptor mediating the interaction of activated platelets and endothelial cells with leukocytes. Platelets release various growth factors (e.g., TGF-β, PDGF, and EGF) that influence endothelial cell physiology and vice versa and activate endothelial signalosome signaling (24).

## Platelet Interaction With the Adaptive Immune Response

Platelet interaction with leukocytes can facilitate the activation of adaptive immune responses. Platelets can promote dendritic cell maturation and NK cell and monocyte/macrophage responses, which themselves affect specific T and B cell responses. Furthermore, platelets can directly affect B cell isotype switching and CD8^+^ T cell proliferation (25). Czapiga et al. demonstrated that platelet-derived CD40L induces the maturation of immature dendritic cells, professional antigen-presenting cells, via the upregulation of co-stimulatory molecules and IL-12/p40 production (26). Kaneider et al. showed that platelets trigger dendritic cell maturation independently of cyclo-oxygenase-derived arachidonic acid metabolites by mechanisms involving CD40L (27). Lastly, our data indicate first that platelets secrete a soluble dendritic cell-activating factor that was shown not to be sCD40L, as was expected from previous *in vivo* and *in vitro* studies, but instead a nucleotide, and second, that cell-to-cell contact does not induce the maturation of dendritic cells, possibly since nucleotide release by platelets was prevented by direct contact with dendritic cells (28, 29).

Adhesive interactions between platelets and monocytes deliver specific signals that initiate inflammatory gene expression, as described by Dixon et al. (30), showing that activated platelets induce COX-2 synthesis in monocytes by signaling at the transcriptional and post-transcriptional levels (30). Moreover, the formation of platelet-monocyte complexes and the detection of platelet-bound CX(3)CL1 on inflamed smooth muscle cells suggest that the CX(3)CL1-CX(3)CR1 axis contributes significantly to platelet and monocyte concentration in atherosclerotic arterial injury (31). Wong et al. (32) showed that platelets interacted with Kupffer cells in the liver sinusoids and that those interactions quickly changed to firm adhesion after specific microbes were captured by Kupffer cells. Elzey et al. reported that platelet-derived sCD40L increases serum IgG levels and germinal center formation under conditions where antigen-specific CD4^+^ T lymphocyte amounts are limiting (33). Regarding T lymphocyte activation and platelets, platelet reduction was shown to decrease intrahepatic accumulation of virus-specific cytotoxic T lymphocytes (CTLs) and organ injury in mouse models of acute viral hepatitis. Moreover, activated platelets contribute independently of their procoagulant function to CTL-mediated liver immunopathology (34). Zamora et al. investigated the proliferation and cytokine release of CD36^+^ CD4^+^ lymphocytes. Flow cytometric analysis and immunofluorescence microscopy indicated that CD36^+^ platelets were responsible for CD36 recognition on CD4^+^ lymphocytes. Moreover, Zamora et al. described that IL-17 and IFN-γ production was reduced in CD4^+^ lymphocytes with bound platelets (35). Furthermore, CD40L-positive T lymphocytes stimulated platelet activation through a CD40-dependent interaction with RANTES release, which activated endothelial cells, and facilitated T cell recruitment (36). Chapman et al. in Craig Morrell's laboratory at the University of Rochester, provided evidence that murine and human platelets express MHC class I molecules and that platelets activate T cells in an MHC class I-dependent manner. This interesting report suggests a novel hypothesis that platelets participate in the initiation of the acquired immune response (37).

## Platelets as Sensors in Innate Immunity in Response to Infectious Stress

It is becoming increasingly clear that platelets have inflammatory functions and can influence both adaptive and innate immune responses. Below are discussed some of the mechanisms by which platelets contribute to the innate immune response.

Platelets express the transmembrane protein CD40 ligand (CD40L, CD154), a member of the TNF receptor family. CD40L engages CD40, a second member of the TNF receptor family also present on B cells, monocytes, macrophages, carcinoma cells, dendritic cells, Kupffer cells and vascular endothelial cells, as well as on non-hematopoietic cells such as endothelial cells, smooth muscle cells, fibroblasts and keratinocytes (38). Platelets also express CD40. CD40 and CD40L are instrumental in both innate and adaptive immunity, with complex functions. Platelet activation leads to the surface expression and secretion of a wide range of proteins. P-Selectin (CD62P) is present on the inner leaflet of platelet granules and, following exocytosis, is expressed on the external leaflet of the plasma membrane. The ligand for CD62P is P-selectin glycoprotein ligand-1, which is expressed on a variety of leukocytes, notably neutrophils, eosinophils, lymphocytes, and monocytes. The GPIIbIIIa complex is present on the surface of quiescent platelets in an inactive, closed configuration (36). However, upon activation, there is a structural change in GPIIbIIIa to an active open state, which allows its binding to ligands. Although the natural ligands for GPIIbIIIa are von Willebrand factor and fibrinogen, the HIV surface protein gp120 can be bound by GPIIIa (along with other receptors) on platelets.

TLR adapters and signaling proteins downstream of TLR activation are potential targets for therapeutic drugs in eukaryotic cells (2); however, a more complete understanding of the platelet signaling complex is necessary. An increasing number of studies report both that platelets participate in the inflammatory process and that they may have an impact on pathogen clearance and the pathogenesis of bacteraemia, sepsis and, potentially, severe sepsis (39–41). However, a recent study presents the opposite view, in which a dual-track clearance mechanism balances innate and adaptive immunity during bacteraemia. Liver macrophages mediate fast clearance of intravascular *Listeria. monocytogenes* via scavenger receptors, in contrast to platelets, whose binding shifts *L. monocytogenes* clearance from “fast” to CRIg-dependent “slow” clearance pathways (42). Of critical importance to immunity and inflammation are Toll-like receptors (TLRs). TLRs are sensors of pathogen-associated molecular patterns (PAMPs), molecular determinants generally expressed by pathogens, specifically infectious pathogens. Several groups have described the presence and functionality of TLRs in mice and humans on both the membrane (TLR2/TLR1/TLR6/TLR4 and TLR9) and within platelets (TLR9); and TLR3 and TLR7 have also been identified (11–13, 43–48). Several recent studies suggest that TLR2,-4 and -9 are targets for bacterial-platelet interactions during severe sepsis and that they provide interesting targets for pharmacological analysis. Clark et al. suggested platelet TLR4 to be a threshold switch for bacterial trapping in severe sepsis. LPS-activated neutrophils, in combination with TLR4-activated platelets, were found to lead to the formation of neutrophil extracellular traps (NETs), which were able to ensnare bacteria in the blood flow for targeting immune clearance events (46). The addition of septic plasma but not control plasma to healthy neutrophils and platelets in the presence of DNA dyes evidenced the formation of NETs. Moreover, the author showed that platelet TLR4 mediates NETosis by decreasing the neutrophil DNA release time from 2 to 3 h (generally observed when neutrophils are stimulated) to ~10 min (when platelets are present). Therefore, it has been suggested that inhibition of platelet activation with TLR4 inhibitors, such as eritoran, may reduce NET formation and limit tissue damage (46). Evidence is also emerging that certain TLRs play a major role in the pathogenesis of infectious and/or inflammatory diseases (49). Sabroe et al. reported that stimulation by natural ligands of TLR2 (Pam3CSK4) or TLR4 (LPS) did not cause any changes in platelet aggregation, the surface levels of CD62P or the intra-platelet calcium levels (44). These data are consistent with the absence of direct effects on platelet activation as a result of the engagement of TLR2 or TLR4; the authors therefore concluded that these receptors are non-functional residues from megakaryocytes. Similar data were obtained by Jayachandran et al. (50) who showed that LPS did not affect platelet responses. In contrast, other groups, including our own, have found that the TLRs on platelets are functional and that their engagement evokes a variety of platelet responses (1, 2, 36, 40). In contrast to other cell types involved in immune responses (e.g., macrophages and dendritic cells), any possible link formed by platelets between innate immunity and adaptive immunity has yet to be proven (1–3, 33, 36, 40). Recently, Panigrahi et al. showed that physiological platelet agonists, primed by either suboptimal concentrations of thrombin receptor-activating peptide (TRAP) or the weak agonist ADP, act synergistically with TLR9 ligands by inducing TLR9 expression on the platelet surface and that the platelet TLR9 receptor is a functional receptor linking oxidative stress, innate immunity, and thrombosis (12). Thon et al. further demonstrated that TLR9 is located in a newly identified intracellular compartment in platelets and described a new organizational and signaling mechanism for TLR9 in human platelets (11). Finally, Hally et al. observed that platelet TLR9 expression was significantly elevated in subjects with acute coronary syndromes (ACSs) compared to that in healthy subjects, which may result in increased sensitivity to TLR9 agonists. Platelet activation caused increased expression of TLR9 in healthy platelets. We suggest that platelet activation, which occurs as part of ACSs, is a potential mechanism explaining the increased expression of platelet TLR9 observed in ACS patients (51). We provided evidence for differential signaling in platelets exposed to various TLR ligands leading to cytokine and chemokine secretion (52, 53). This difference indicates that platelet TLRs are functional, as they not only engage intracellular signaling pathways but also select among distinct adaptors (MyD88 vs. TRIF) to terminate NF-kB phosphorylation. The correlation between platelet TLR2 and TLR4 stimulation *in vitro* and the NF-kB/TRIF/Myd88 adaptor and signaling molecules is currently under investigation. In addition, studies showing the expression of TLR3 (54) and TLR7 (48) in human platelets have been published recently. Human platelets express TLR3 and are able to respond to poly I:C, indicating that these cells influence the innate immune response after exposure to viral dsRNA (54). Encephalomyocarditis virus (EMCV) infection rapidly reduces platelet count, and this phenomenon is credited to platelet Toll-like receptor 7 (TLR7) (48). Interestingly, Koupenova et al. demonstrated that platelets express all TLR transcripts and that these transcripts are more important in women with regard to cardiovascular risk and inflammatory markers (55).

Platelets are the major physiological contributor of sCD40L in plasma (56, 57). As outlined above, these soluble molecules can interact with the epithelium lining cells or mononuclear circulating cells that constitutively express CD40 counter receptors. It has been suggested that platelets can alter the binding of CD40/sCD40L, which is essential to inflammation. Platelets are cells that co-express surface CD40 and sCD40L molecules (58–63) in a platelet activation-dependent manner (CD40L is expressed and secreted only after activation, unlike CD40, which is constitutively expressed and not upregulated). However, while sCD40L is characteristic because of its quantitative and qualitative importance, this molecule is just one of the many secretory platelet molecules that contribute significantly to both hemostasis and immune modulation. In addition to releasing molecules that alter immune responses, platelets are involved in antimicrobial responses; indeed, platelets aggregate when exposed to certain bacteria (such as *Staphylococcus aureus*) and viruses (such as HIV), which may trigger responses to danger signals (39, 40, 64). In support of this finding, platelet degranulation, endocytic bacterial, and viral engulfment, and the release of antibacterial/antifungal proteins have been observed in conjunction with platelet aggregation events. Several studies have attempted to determine the involvement of platelets in immune responses dependent on CD40/CD40L and to determine the interactions between platelets and peripheral B cells. Platelets and B cells (in an *in vitro* co-culture model) were mutually activated, as validated by the increased expression of membrane platelet CD62P and B cell CD86. Platelet and B cell interactions were accompanied by changes in the membrane expression of CD40 and CD40L by both platelets and B cells. Differentiated B lymphocytes increased their production of IgG1, IgG2 and IgG3 but not IgG4, IgA, or IgM after a 3-days incubation with platelets *in vitro* (65). Another example of indirect interactions is at the interactions between platelets and macrophages in innate immunity and inflammation (66).

Platelets respond rapidly to changes in their environment, as they express surface receptors for a variety of ligands, such as the subendothelial proteins von Willebrand factor and collagen, as well as soluble agonists, such as thrombin, ADP and TxA2. This activation process leads to a variety of changes in platelets, including the extension of pseudopodia, the secretion of granule contents and PMPs, the synthesis and secretion of TxA2 and IL-1β, the formation of a procoagulant surface and the surface expression of a range of adhesive proteins, either by the exposure of granular membrane proteins on the plasma membrane or by structural changes in surface proteins from inert to active conformations (3).

While it is not yet generally accepted that platelets are also stimulators of immunity and inflammation, several recent reports argue in favor of acknowledging platelets as sensors in innate immunity and players in inflammation (2, 67–71). Platelets exert immune functions by acting to remove pathogen-infected host cells by binding directly to bacteria, viruses, and fungi (1, 39) and by mediating interactions between target cells and these infectious agents to potentiate the immune response (3, 33, 72). As a consequence, platelets have been linked with various inflammatory pathologies, such as cardiovascular disease (73), sepsis (72), and arthritis (36). A variety of mechanisms are involved in the contribution of platelets to the inflammatory process, including the increased expression of receptors for various immune mediators such as cytokines and chemokines and the exocytosis of a range of soluble factors, immunomodulatory factors, growth factors, biological response modifiers, etc., from α-granules (74–79). Several proteins are associated with the inflammasome family. These proteins are divided into two groups depending on the domains they contain: NLRPs contain pyrin domains and NLRCs contain caspase recruitment domains (CARDs). Hottz et al. noted that platelets constitutively express the inflammasome components NLRP3 and ASC (apoptosis-associated speck-like protein containing a caspase recruitment domain) and can use them to assemble functional inflammasomes, activate caspase-1, and process IL-1β (80).

In addition, in patients with dengue or after platelet exposure to dengue virus *in vitro*, increased expression of IL-1β in platelets and platelet-derived microparticles was observed. Infection with dengue virus results in NLRP3 inflammation, caspase-1 activation, and caspase-1-dependent IL-1β secretion. IL-1β derived from platelets is released mainly as microparticles through mechanisms dependent on inflammatory NLRP3 triggered by mitochondrial ROS. Activation of IL-1β-rich microparticles by the inflammasome and platelet shedding is correlated with increased vascular permeability. These findings show that platelets contribute to the increased vascular permeability in dengue virus infection by the inflammation-dependent release of IL-1β (80).

Moreover, Dr. Craig Jenne group are interested by the infections mediated by multidrug-resistant *S. aureus*. Recently, Surewaard et al. demonstrated (81), using an elegantly intravital imaging, that alpha toxin targets platelets directly, resulting in circulation detrimental aggregation. Moreover, neutralizing alpha toxin during infection of *S. aureus*, while escaping microvascular damage, does not interfere with beneficial platelet responses. In this context, Platelets are always able to recruit macrophages and participate to the eradication of *S. aureus*. Considered platelets as sensors in innate immunity in response to infectious stresses (1, 2, 36, 39, 82–94) contributes to the understanding of the interrelationship between infection, inflammation, and coagulation.

## Platelets as Sensors of Storage Lesions as a Non-Infectious Stress

Platelet concentrates for transfusion are living cell products with a certain life span that degrade in a physiological mechanism-dependent manner via mechanisms that may be accelerated by mechanical production and storage mechanisms (95). Platelets prepared for transfusion are subject to stress injury upon collection, preparation and storage (96). Under these types of stress, platelets undergo morphologic/metabolic changes likely to lead to platelet activation and an increase in the concentration of BRMs (82). *Ex vivo* platelet processing can have an effect on BRM secretion (97). These BRM-promoting events lead to negative changes and a gradual deterioration in platelet viability, structure, and function.

When stored as PCs, platelets can undergo changes that are mainly related to the storage solutions and conditions (platelet agitation and storage temperature and time).

In general, PCs—especially those prepared for prophylactic usage—are stored for an average of 5 days at a maximum temperature of 22 ± 2°C under constant, gentle agitation to prevent platelet aggregation. Additionally, buffy coat-derived pooled platelet concentrates (PPCs) and single-donor apheresis platelet concentrates (SDA-PCs) are stored in suspension in 35% donor plasma and 65% platelet additive solution (PAS). Compared to platelet storage in autologous plasma, platelet storage in an additive solution has satisfactorily improved platelet function preservation. PASs are generally used as plasma replacements to (i) reduce the quantity of plasma transfused; (ii) avoid the transfusion of large volumes of plasma to reduce the incidence of adverse reactions and circulatory overload; (iii) enable certain photochemical treatments for pathogen inactivation; and (iv) maintain storage conditions (98). Platelet storage lesions include the appearance of platelet morphological changes, activation markers, GPIbα expression loss, α granule secretion, and mitochondrial dysfunction (99). Platelet concentrate storage can lead to the secretion of several BRMs, such as sCD40L, PDGFAA, RANTES, IL1β, IL6, IL7, IL8, PF4, IL13, OX40L, IL27, and TGFβ (2, 82). Generally, extended PC storage is accompanied by increased BRM production, which may be related to an increase in the percentage of adverse events (AEs) observed according to PC storage time. To minimize AEs, it would be preferable to transfuse PCs as early as possible. It is fitting, however, to consider this conclusion in light of the PC production and issuing constraints on blood establishments according to the demand for the product in hospital banks. In particular, it has been demonstrated that from the 3rd day of PC storage, there is a significant increase in the concentration of BRMs, especially sCD40L (96). These observations suggest that storage lesions play a role in the inflammation caused by PC. sCD40L induces the production of reactive oxygen species (ROS) during PC storage, leading to an increase in the production and release of pro-inflammatory substances (100).

Additionally, the type of PC processing used during the preparation and storage process can have an effect on platelet activation. Leitner et al. showed that platelets stored in an InterSol™ solution exhibited significantly higher initial activation levels, as indicated by CD62P expression, than platelets stores in other additive solutions (Composol® and SSP^+^®) (101). However, platelet storage in an additive solution has demonstrated a certain number of benefits, especially a reduction in serious adverse reactions (102). Although the different types of PCs are of comparable quality, there is debate about their safety. Daurat et al. showed that AEs were less commonly related to PPCs than to SDA-PCs (103). These results challenge the widespread use of SDA-PCs and suggest that these concentrated should be prescribed for specific indications. Evaluation of the risk/benefit balance of transfusing different types of PCs would enable the prescription of the optimal product according to the medical indication.

BRMs contained in PC are also transfused. It has been shown that BRMs can induce immune responses (33) and post-transfusion reactions (104) and can affect hemostasis (105) and inflammation in the recipient (106). Storage lesions triggered by extrinsic factors (preparation methods) or intrinsic mechanisms (plasma and platelet factors, residual leukocytes) could be largely responsible for both reducing the therapeutic efficacy of PC transfusion and inducing AEs (76). In addition to blood platelets, BRMs contained in PC are also transfused to the recipient. Among these molecules, sCD40L is described as being partly responsible for febrile non-hemolytic transfusion reactions (FNHTRs) after platelet transfusions (58, 107). In addition to its role in inflammation, CD40L seems to play a role in AEs. sCD40L is found in PCs, and its concentration increases during storage (96). Numerous studies have shown that sCD40L is involved in PC transfusion reactions (106, 108). In addition, we showed that other soluble factors, such as IL27 and sOX40L, are involved in FNHTR (104). Several soluble factors with high predictive value for the occurrence of AEs, such as sCD40L, IL13, and MIP1α, have been identified, primarily via machine learning algorithms (109). Indeed, this study shows a correlation between the concentrations of sCD40L and IL13 and the onset of AEs. Additionally, the concentration of MIP1α found in the supernatants that induced AEs seems to be able to differentiate the type of AEs, FNHTR or allergies. PCs also contain mitochondrial DNA (mtDNA), which is associated with adverse effects (96, 110). Boudreau et al. showed that activated platelets release mitochondria, in both encapsulated microparticles and membrane-free organelles. Extracellular mitochondria are found at higher levels in transfused PCs that caused acute reactions (FNHTR, cutaneous, and cardiovascular signs) in transfused patients than in those that did not (99, 110, 111).

It is clearly acknowledged that the increased levels of cytokines and chemokines in platelet concentrates developed during storage, in the absence of detectable exogenous stimuli, can contribute to AEs (96, 112). In addition to cytokines/chemokines, platelet Extracellular vesicles (EVs), and platelet microparticles (PMs), which are important mediators of inflammation and immune response regulation also seem to be involved in the onset of AEs (113, 114). EVs are a heterogeneous group of structures and comprise a large group of particles, including exosomes and microvesicles, and are released from virtually all cell types. EVs can be divided according to their size into microparticles (MPs) or microvesicles (MVs) that vary in size between 0.1 and 1 μM, and exosomes in size of 30–100 nm (115). Platelet exosomes strongly expressed tetraspanin CD63, CD9, CD63, TSG101, ALIX, CD31, CD41, CD42a, P-selectin, PF4, and GPIIb/IIIa. Platelet exosomes might play a lesser role in procoagulant activity than PMPs. Platelet exosomes can directly stimulate target cells by providing ligands that increase the secretion of various signaling molecules, e.g., growth factors or cytokines. They can also transfer membrane receptors and molecules of adhesion. In addition, Platelet exosomes provide proteins, mRNA, and transcription factors that cause target cell epigenetic reprogramming (116).

PMPs containing microRNA can also be involved in a pathophysiologic response and AE induction following PC transfusion. Additionally, studies have shown that pathogen reduction technologies aimed at reducing the potential risk of transfusion-transmitted infections induce platelet activation and a reduction in the mRNA (117) and microRNA levels (118). These RNA changes are correlated with an increase in the PMP concentration. As a result, it seems likely that pathogenic agent reduction technologies can increase PMP formation in PCs (118). Given the pro-inflammatory properties of PMPs, it is reasonable to presume that they can aggravate acute and chronic inflammatory reactions in blood vessels, such as those associated with platelet transfusion and atherosclerosis (119).

## Conclusion and Future Directions

**Figure 1:**
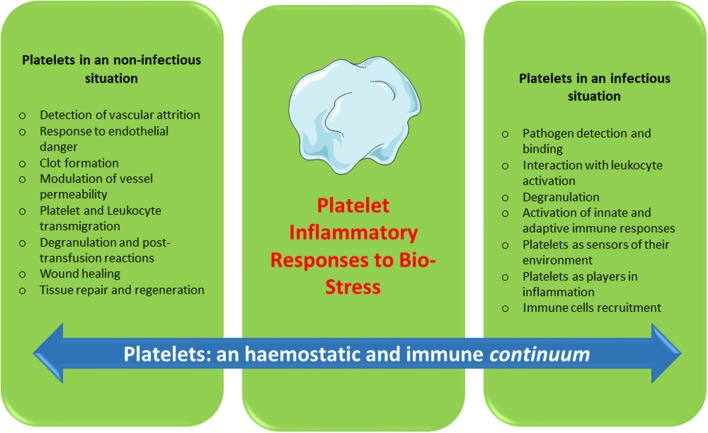
Platelets: an hemostatic and immune continuum.

Platelets contain and secrete numerous cytokines and other immunomodulatory proteins, which may be candidates for innovative targeted therapeutic approaches. For instance, there is now sound evidence that the platelet-activating factor (PAF)/PAF-receptor pathway is a promising target for pharmacological involvement in acute coronary syndrome (120), central nervous system diseases (121), autoimmune diseases (122), and rheumatoid arthritis (123). Platelet-related CD40L, IL-1β, PF4, and RANTES are currently under consideration as molecular targets against inflammation and hypercholesterolemia syndromes (124). Platelets continue to motivate much biological research. To date, a substantial amount of data has been collected over several decades on the hemostatic properties and immunogenic competence of platelets, yet much about the roles of platelets in physiological and pathological processes remains to be clarified. Furthermore, if platelets are immune sentinels with the capacity to bridge innate and adaptive immunity, preformed products from platelets, and products resulting from platelet neosynthesis can act on cell-cell interactions (platelet-dendritic cell, platelet-B cell and platelet-T cell) and play a major role in immune responses. Several reports have investigated “platelet physiology” as an immune cell concept (2, 71), and a notable number of papers (67–70, 90, 125, 126) have recently detailed this new concept. Indeed, future directions for research concern the critical role of platelets as an immune cell in the host immune response. It is now clear, therefore, that in addition to their roles in hemostasis and thrombosis, platelets have a large range of other functions, notably playing key roles in the inflammatory process, immune responses (2, 82, 84), regenerative medicine and host defense against pathogens (Figure 1). The challenge for therapeutic intervention in pathological processes will be to identify drugs that block specific targets involved in the complex contribution of platelets to inflammation/immunity without affecting their hemostatic function.

## Author Contributions

FC, SL, PB, TB, HM, PM, OG, and HH-C have made a substantial, direct and intellectual contribution to the work, wrote and approved it for publication.

### Conflict of Interest Statement

The authors declare that the research was conducted in the absence of any commercial or financial relationships that could be construed as a potential conflict of interest.
